# Bridging the Divide: Brain and Behavior in Developmental Language Disorder

**DOI:** 10.3390/brainsci13111606

**Published:** 2023-11-19

**Authors:** Noelle Abbott, Tracy Love

**Affiliations:** 1 School of Speech, Language, and Hearing Sciences, San Diego State University, San Diego, CA 92182, USA; ntodd6857@sdsu.edu; 2 San Diego State University/University of California San Diego Joint Doctoral Program in Language and Communicative Disorders, San Diego, CA 92182, USA

**Keywords:** developmental language disorder, child language disorders, language processing, neuroimaging, MRI, brain–behavior relationship, theoretical accounts

## Abstract

Developmental language disorder (DLD) is a heterogenous neurodevelopmental disorder that affects a child’s ability to comprehend and/or produce spoken and/or written language, yet it cannot be attributed to hearing loss or overt neurological damage. It is widely believed that some combination of genetic, biological, and environmental factors influences brain and language development in this population, but it has been difficult to bridge theoretical accounts of DLD with neuroimaging findings, due to heterogeneity in language impairment profiles across individuals and inconsistent neuroimaging findings. Therefore, the purpose of this overview is two-fold: (1) to summarize the neuroimaging literature (while drawing on findings from other language-impaired populations, where appropriate); and (2) to briefly review the theoretical accounts of language impairment patterns in DLD, with the goal of bridging the disparate findings. As will be demonstrated with this overview, the current state of the field suggests that children with DLD have atypical brain volume, laterality, and activation/connectivity patterns in key language regions that likely contribute to language difficulties. However, the precise nature of these differences and the underlying neural mechanisms contributing to them remain an open area of investigation.

## 1. Introduction

Developmental language disorder (DLD) is a heterogenous neurodevelopmental disorder that affects a child’s ability to comprehend and/or produce spoken and/or written language but cannot be attributed to hearing loss or overt neurological damage (coded in the ICD-11 §6A01.2). In recognition of the growing preference for the term DLD, we have chosen to use it for this review instead of specific language impairment (SLI) or developmental dysphasia, though the research referenced throughout includes publications that use both terms. DLD affects around 7% of children in the US making it more prevalent than other neurodevelopmental disorders, such as autism spectrum disorders (ASD) and dyslexia [1,2,3,4]. Moreover, adults who were diagnosed with DLD as children often experience anxiety and depression and tend to struggle with social relationships, preferring environments and vocations that do not require strong language and literacy skills [5,6]. Despite the prevalence and profound life-long impact DLD can have on a person, little is understood about the neurological basis or etiology of the disorder or how observed language impairments arise.

DLD is typically diagnosed after the age of 4 (around the time a child enters into preschool), when it becomes clear that the child has fallen behind their same age peers in terms of receptive and expressive language skills [7]. Yet, it is likely that the neural substrates underlying the disorder are in place prior to receiving a diagnosis. Current research suggests that some combination of genetic and environmental factors influence neural development in this population, but it is unclear if aberrant brain pathology causes DLD or if DLD leads to altered brain structure and function [8]. Further, there is significant debate regarding theoretical accounts of language impairment patterns observed in children with DLD. To date, none of the neurological or theoretical explanations of DLD fully account for the range of symptoms across individuals or the differing results across research studies [9]. This disconnect has resulted in some researchers defining DLD as a heterogenous disorder that may actually be a spectrum disorder with different phenotypes, like ASD, or may even exist on the same continuum as ASD [10,11,12].

Core to this paper is the notion that the term heterogeneity is oftentimes misused to describe the differences found across studies that are better attributed to differences in research design (population identification, task demands, etc.). For example, if a study uses an assessment that poorly identifies children who have DLD (i.e., low sensitivity) compared to those who do not (i.e., low specificity), that could lead to the DLD group appearing to have more variability in measured behaviors [13,14]. Task demands may also influence how heterogeneous the DLD group appears, especially if the comparison group is poorly matched on age or other criteria. When studies accurately measure known areas of difficulty for children with DLD, such as morphosyntax, they in fact often perform similarly to one another (i.e., heterogeneity is reduced) [14]. In this overview, we broach the topic of heterogeneity briefly to suggest that children with DLD struggle with a range of linguistic and non-linguistic behaviors, but we do so with the knowledge that within specific domains of language they show more consistent impairment patterns than their typically developing peers. Table 1 outlines language problems commonly reported in children with DLD.

### Purpose

The careful characterization of heterogeneity in DLD is important to note because differing patterns of results for both behavioral and neuroimaging studies have obscured what may otherwise be true differences between children with DLD and TD children. As a result, findings have not been synthesized in a way that moves the field forward. As such, though we recognize that there are differences in outcomes across studies, here the aim of this overview is to shift the focus away from addressing outcome differences across studies to identifying converging evidence, so that we can begin to bridge the theoretical and imaging fields. While a few other overviews exist (see [16,17,18]), none that we are aware of link neuroimaging patterns with various theoretical approaches that attempt to capture the range of language impairment patterns found in DLD.

We approach this by first reviewing the underlying neuroimaging patterns to date in DLD (structural (Section 2) and functional (Section 3)). We then summarize a subset of common theoretical accounts of language impairment patterns (across production and comprehension) while attempting to bridge the gap between neuroimaging literature and theory to elucidate brain behavior connections in DLD (Section 5 and Section 6). As will become clear from this overview, the link between theoretical accounts of DLD and findings from neuroimaging research is based on limited evidence; therefore, we will conclude by proposing future directions for neuroimaging research to better understand how aberrant brain structure and function relates to observed language impairments in DLD (Section 7).

## 2. Structural Neuroimaging Findings in DLD

In this section and in Section 3 below, we describe the structural and functional outcomes of neuroimaging studies as a way to set up the integration of neuroimaging findings in support of theoretical models of language in DLD (Section 5, below).

The link between brain development and language outcomes in children with DLD is unclear, and this lack of connection is apparent when reviewing the DLD neuroimaging literature. Over the past 50 years, there have been fewer than 60 neuroimaging studies (excluding EEG studies) with children diagnosed with DLD. The majority of these studies have focused on structural brain differences when compared to language-unimpaired (neurotypical) children or children with other neurodevelopmental language disorders, such as children diagnosed with ASD and concomitant language impairment. Though there are some consistencies that will be discussed below, it is important to note that the picture portrayed here is tenuous at best due to the limited number of studies that confirm these consistent findings as compared to the larger number of studies that have contrasting results. In this paper, we determined that differences in participant selection and inclusion, diagnostic criteria, methodology, and analyses used underlie the disparate findings to date (see Appendix A, Table A1). As such, comparing the results across studies and evaluating how structural and functional brain abnormalities contribute to language impairment in children with DLD is challenging. Nonetheless, in this section and in Section 3 below, we provide a general overview of structural and functional neuroimaging findings in DLD and highlight consistent patterns of results. Additionally, when appropriate, we link findings to patterns found with other language-impaired populations to provide credence to the structural and functional patterns found in DLD.

### 2.1. Structural Brain Differences

Across development, the human brain undergoes a wide variety of structural changes in order to support increasing cognitive demands and the acquisition of new skills [19]. The emergence of white matter pathways in the brain begins in utero following formation of the neural tube and production and migration of neurons [20,21]. The brain then continues to differentiate and refine following birth. Infancy and early childhood are periods of rapid brain development, where the myelination of axons and synaptic reorganization and pruning are abundant in order to strengthen neuronal populations that frequently fire together as well as support those neuronal networks associated with new skills [20,21]. As a result of these changes, the gross structure of the brain continues to visibly change within the first few years of life, with subtle decreases in gray matter volume and increases in white matter volume up until early adulthood [22]. Thus, changes in individual neurons as well as neural networks impact gross brain structure across development.

Any perturbations to the tightly orchestrated processes that contribute to brain development can contribute to a range of developmental disorders [23]. In fact, across different neurodevelopmental disorders, there is widespread evidence of volumetric brain differences compared to neurotypical peers [24,25,26,27,28,29]. For example, individuals with ASD have been shown to have whole and regional brain volume differences when compared to age-matched control children. Lange et al. (2015) found that young children with ASD had larger overall brain volumes than their typically developing peers but as they aged, they showed atypical regional volume decreases [30]. Findings such as these underscore the importance of investigating brain differences between typically and atypically developing populations, so that we can begin to uncover how structural (and functional) alterations contribute to language impairment. In the sections that follow, we provide an overview of structural brain differences in DLD starting with global brain volume, moving to a discussion of gray and white matter volume and integrity. It should be noted that the structure of this overview should not be viewed as an annotated summary of findings, but instead presents thematically related information based on the broader categories that were just described.

#### 2.1.1. Global Brain Volume

Studies investigating measurements of global brain volume (i.e., the whole brain, the hemispheres, and the cerebral lobes) in children with DLD have found differences when compared to neurotypically developing (TD) children, suggesting that like other neurodivergent populations, there are abnormalities in brain structure that are associated with the disorder. In typical development, total cerebrum volume and surface area increases from birth until reaching its peak at around 11–12 years of age [31]. The few studies that have reported on these metrics in DLD point to children with DLD having smaller overall brain volumes, hemispheres, and cerebral lobes than typically developing children [32,33,34,35]. However, other studies, such as Herbert et al. (2003), have found contrasting results (e.g., larger brain volumes compared to TD children) [36]. These discrepant findings may be due to differences in methodological choices (e.g., voxel-based vs. semi-automatic brain morphometry). Another possibility to account for differences reported here, as well as throughout this paper, is that children with DLD may have different brain volumes relative to their TD peers at different stages across development, since DLD is indeed a developmental disorder. Therefore, studies aimed at measuring longitudinal changes in DLD (similarly to Lange et al.’s 2015 [30] approach with ASD) are needed as they will help elucidate differences in measures of global brain volume across development.

#### 2.1.2. Total Gray Matter Volume

Across the human lifespan, total gray matter volume increases in utero until its peak around 6 years of age followed by a non-uniform decrease from late childhood to late adulthood [31]. Like with overall brain volume, studies investigating total gray matter volume in children with DLD have inconsistent findings because they employ different methods (e.g., volume-based vs. surface-based analysis) and their participant groups (for both DLD and TD children) rarely overlap in age, demographics, and inclusionary/exclusionary criteria (e.g., prior neurological history, assessment scores, etc.). As we focus this paper on converging findings, one common pattern is that reductions in overall gray matter volume (compared to TD children) are evident in younger children with DLD but diminish as they age. Soriano-Mas et al. (2009) used voxel-based morphometry (VBM) to examine gray matter differences in two groups of children with DLD, younger children under 12 years of age (*n* = 19), and older children over 12 years of age (*n* = 17) [37]. They found that when looking at the DLD group as a whole, they had greater global gray matter volumes than TD age-matched controls, but when they separated participants by age group, the younger children with DLD had greater gray matter volumes than control children of the same age, while the older children with DLD showed no differences in gray matter volume compared to their neurotypical peers. Consistent with Soriano Mas et al.’s findings that morphological differences in DLD diminish with age, Badcock et al. (2012) found no differences in overall gray matter volume between older TD children and age-matched children with DLD (mean age: 13.5) [38]. Typically, total cerebrum volume, surface volume, and subcortical volume reach their peaks around twelve years of age, with total gray matter volume peaking earlier, followed by a slow decline [31]. It may be the case that the proportion of gray matter in TD children after six years of age decreases enough to match that of children with DLD at twelve years of age, while overall brain volume differences remain detectable between groups across late childhood and adolescence. These findings align well with the literature from other neurodevelopmental disorders, such as ASD, that report amplified volumetric differences in younger age groups that eventually diminish around 9–11.5 years of age [39].

As described above, maturational differences underscore the importance of carefully considering the age range of participants included, but other methodological differences, such as not accounting for overall brain size within participants, can also impact results. Larger brains have more brain tissue and can give the appearance of increased gray matter, when in reality, the amount of gray matter may be proportional to overall brain size. To account for proportional differences in gray matter volume, Girbau-Massana et al. (2014) included intracranial volume (ICV) as a covariate in their analyses [33]. Unlike Soriano-Mas et al.’s (2009) study [37], which found that younger children with DLD had larger gray matter volumes, Girbau-Massana et al. (2014) [33] found that the young children with DLD had *lower* overall gray matter volumes than their TD peers. The discrepant findings again may be related to accounting for ICV or they could be due to the fact that 6 of the 10 children with DLD in their study were also diagnosed with a concomitant reading disorder. Regardless of the inconsistent findings, there is limited evidence indicating that children with DLD as a whole have abnormalities in gray matter volume. What remains unclear is the source of gray matter differences; that is, does it represent a difference in the overall surface area or is it due to differences in cortical thickness? Both cortical thickness and surface area follow different developmental trajectories, suggesting that they are driven by partially distinct processes [40]. In the only large-scale study investigating these metrics in DLD, Bahar and colleagues (2023) found that children with DLD (ages 10–16 years old) had lower measures of surface area and, to a lesser extent, volume, but not cortical thickness, when compared to their TD peers [41]. Changes in the surface area of the brain are associated with gyrification (or the folding of brain tissue), which is related to more efficient neural processing [42]. Based on these findings, it is possible that differences in gray matter volume may be related to less efficient neural networks (as indexed by decreased surface area) within language-related brain regions in children with DLD.

In the next section of this paper, we summarize regional gray matter volume differences for areas linked to language function to explore how differences in gray matter volume within language-related brain regions can impact language abilities [43,44,45,46]. We also discuss differences in regional asymmetries as they are a common feature of typically developing brains that support growing functional specializations between the two hemispheres.

### 2.2. Regional Brain Differences

**Figure 1:**
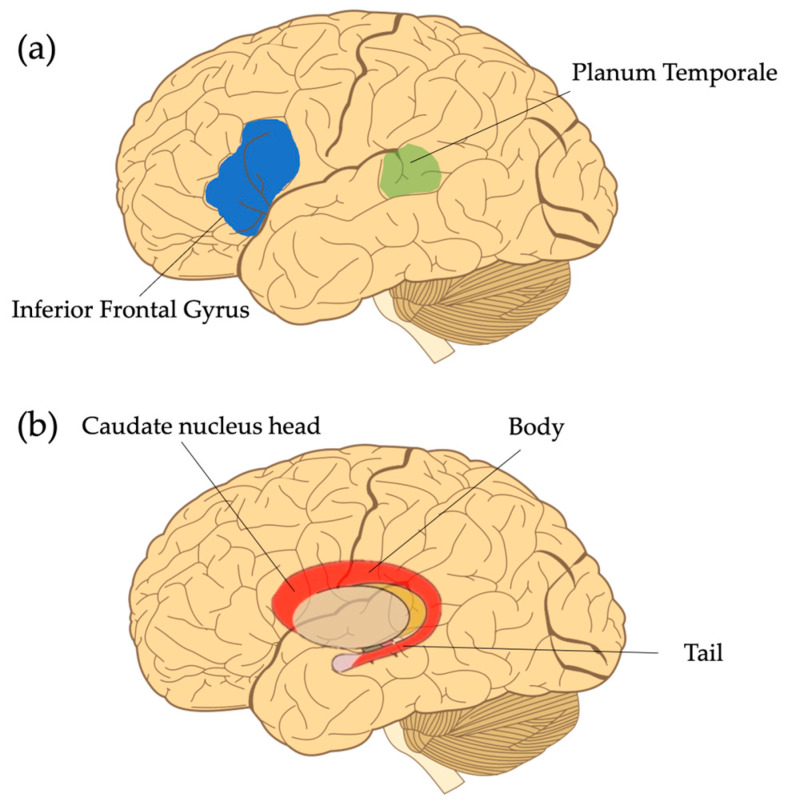
Commonly reported regions of interest in studies investigating structural gray matter differences in DLD. (**a**) The inferior frontal gyrus and the planum temporale. Note that the transparency of the planum temporal is meant to indicate that it is not visible on the lateral surface of the brain. (**b**) The caudate nucleus located subcortically. Note that it can be further subdivided into the head, body, and tail, but will be discussed as a whole in the text. Figure adapted from Hugh Guiney, CC BY-SA 3.0, via Wikimedia Commons https://en.m.wikipedia.org/wiki/File:Human-brain.SVG (accessed on 12 September 2023).

One of the most consistent findings in children with DLD is anomalous gray matter volume and symmetry within the perisylvian language zone. Though across the DLD literature a number of regions within this zone have been discussed [16,17], this section highlights three specific regions which have consistently been shown to have different characteristics in children with DLD as compared to TD children, namely the planum temporale and inferior frontal gyrus (Figure 1a). In addition to these standard language regions, the other region that will be discussed is the caudate nucleus, as it has been theorized to support speech and language processes (Figure 1b).

#### 2.2.1. Planum Temporale

Perception and processing of speech sounds requires the ability to attend to rapidly changing auditory stimuli. The planum temporale is thought to play a key role in this process and has been posited to be a factor in the left hemisphere’s dominance for language [47]. Prior research has indicated that the left planum temporale is comprised of densely packed cortical columns that facilitate processing at higher temporal resolutions [48]. As a result of this left hemisphere specialization, the planum temporale is generally larger on the left than the right in typically developing populations [49,50,51]. However, children with DLD, as well as children from other language-impaired populations, such as those with ASD with language impairment [52] and dyslexia [53], do not exhibit this pattern, suggesting that there is a connection between language impairment and alterations in volume of the planum temporale. 

In children with DLD, the exact nature of these differences is unclear as some studies have demonstrated that they have larger planum temporale in the right hemisphere [22,32,54], while others have found that when compared to controls, the planum temporale size is reduced in the left hemisphere [55,56,57], or equal in size [58]. Differences in methodological approaches in the segmentation of the planum temporale may have contributed to the conflicting outcomes. For example, in a study by De Fossé et al. (2004), the authors noted that they included broader regions (the horizontal and posterior ascending portions) of the planum temporale in their measurements (delineated by a trained technician using anatomical landmarks), whereas other studies only included surface volume measurements based on automatic parcellation methods [59]. Though the studies referenced here contrast in terms of the directionality of differences (likely due to methodological differences), both indicate a potential link between abnormal planum temporale asymmetry and language impairment. 

#### 2.2.2. Inferior Frontal Gyrus

**Figure 2:**
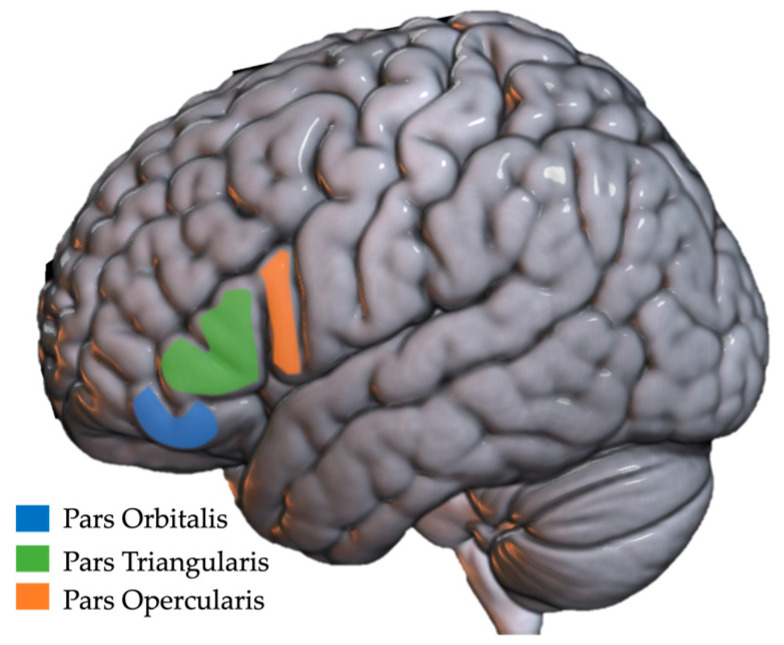
The three subdivisions of the inferior frontal gyrus shown in the left hemisphere.

Another commonly implicated region in children with DLD is the inferior frontal gyrus (IFG). The IFG is comprised of three functional regions, the pars triangularis, the pars opercularis, and the pars orbitalis, though the pars opercularis is sometimes further subdivided into dorsal and ventral regions (Figure 2) [46]. This distinction is important as it can impact structural and functional comparisons across studies, yet studies often report results for the entire IFG or for the region known as Broca’s area (pars opercularis and pars triangularis) rather than the individual components. The functional role of left IFG regions has been a long-standing source of debate amongst researchers (see summary by Rogalsky et al., 2008) [60,61,62]. Though theories differ, they seem to converge on Broca’s area being a language support region and the left pars orbitalis playing a role in covert articulation and semantic processing [63].

In typically developing individuals, the left IFG tends to be larger in the left hemisphere than in the right, which is thought again to reflect left hemisphere language dominance [59]. In children with DLD, the regions within the left IFG have been shown to lack this pattern with some studies reporting that they have more gray matter in this region [38,64,65] and other studies reporting that the left IFG is smaller in children with DLD compared to controls [32,59,66]. Interestingly, of the studies reporting overall smaller left IFG volumes in DLD, some pointed to a group correlation between a smaller left pars triangularis and worse observed language outcomes [32,66]. However, as has been emphasized throughout this paper, the interpretation of findings should be approached with caution as results are often affected by research design. For example, Plante et al. (1991) found that the size and symmetry pattern of the IFG in children with DLD did not differ from age-matched peers as a group, but when looking at individual patterns of symmetry within the IFG, results varied from child to child, with some demonstrating reversed asymmetry while others did not [54]. Plante et al.’s results may be due to maturational effects, given that the participants varied in age from 4 years to almost 10 years of age. Another possibility is that there may be a tradeoff between abnormal development of the left IFG early in life and compensation of other structures in the brain. In support of the latter possibility, Lee et al. (2020) reported individual differences in the size of the left IFG with some children with DLD in their study having increased volume in the right pars orbitalis, which may indicate compensation of the right IFG in those who had abnormal left IFG volumes [64]. More research is clearly needed to understand differences in the left IFG volume and symmetry in children with DLD as well as the functional and structural connection between left and right homologue regions. As pointed out in the planum temporal section above, results converge on a similar theme; abnormal brain patterns in DLD can be linked to observed language impairment.

#### 2.2.3. Caudate Nucleus

Other subcortical regions outside of the cortex, such as the basal ganglia and thalamus, have also received attention in children with DLD, as they connect to other language regions and are thought to play a supportive role in certain aspects of language such as complex syntactic processing, semantic processing, phonological processing, word generation, and more generally, attentional resource allocation [67,68,69,70,71]. Focusing in on one of the structures within the basal ganglia, the caudate nucleus (also referred to as the caudate), some studies have found that it tends to be larger in the left hemisphere than in the right in typically developing individuals and it has projections to other cortical regions involved in verbal working memory, language comprehension and production, and procedural learning [69,72,73]. However, in children with DLD, studies have reported a bilateral reduction in gray matter volume of the caudate, with the exception of Soriano-Mas et al. (2008), who reported an increase in caudate volume in younger children that was not present in older children [35,36,37,38].

The caudate has also been implicated in the neuropathology of the famous KE family, known for their genetic mutation of the FOXP2 gene which, it has been argued, has rendered the language/speech of over half the family members agrammatic (i.e., lacking grammatical features) and unintelligible. In a study specifically looking at the relationship between language impairment and the caudate in this family, Watkins et al. (2002) found that the affected family members had smaller caudate volumes than unaffected family members or age-matched controls, and that the size correlated with nonword repetition abilities [65]. Interestingly, in a study by Krishnan et al. (2022) investigating the macromolecular content of gray matter in the caudate in DLD, they found a reduction in myelin content in the caudate nucleus (as well as the inferior frontal gyrus) when compared to controls and this reduction was related to lower language proficiency [74]. During childhood and adolescence, myelin content increases across cortical gray matter regions, while subcortical regions see less pronounced changes [75]. Further, brain regions associated with higher-order cognitive functions, such as language, require longer periods of myelination and are impacted by not just genes but also the environment [76]. It may be the case that the corticostriatal circuit, connecting regions of the caudate to the frontal lobe, associated with learning, is aberrant in DLD. Though more studies are needed, novel findings about the underlying architecture of commonly implicated brain regions in DLD may reveal important insights into DLD pathology. Given that brain regions do not function in isolation, we now turn our discussion to the white matter pathways that support structural connections between these commonly implicated gray matter regions.

### 2.3. White Matter Pathways

**Figure 3:**
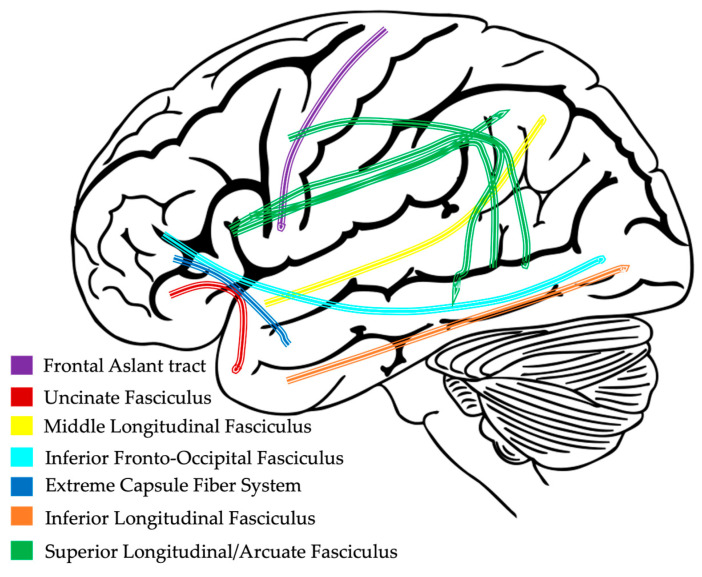
Example of white matter pathways connecting gray matter regions in the left hemisphere.

Historically, investigations into brain function have focused on the size and engagement of cortical and subcortical gray matter regions of the brain. However, a missing piece of those investigations are the connections that allow gray matter regions to coordinate activity. Since we know brain regions do not operate in silos, recent technological advances have provided scientists a way to measure the important connections that exist between gray matter regions, known as white matter, which comprises the structural wiring of the brain (Figure 3). Due to the early development of the brain, white matter pathways are already present by 30 weeks of gestation [77]. However, between birth and two years of age, children undergo a period of rapid brain development, highly influenced by genes and the environment, that helps further shape the neural architecture of the brain [20,78]. As children continue to develop, white matter volume continues to increase until around the fourth decade of life to support improvements in cognitive skills [19,79,80].

There is a strong relationship between neural activity associated with new skills and the formation of efficient, myelinated white matter pathways that connect gray matter regions throughout the brain [19]. As a result of this critical interaction, any deviation in the typical formation of white matter pathways will likely contribute to functional impairments [20]. Prior research has demonstrated a link between white matter alterations and language impairment in children with a range of neurodevelopmental disorders, including autism spectrum disorder, dyslexia and other reading disorders, and epilepsy [81,82,83,84]. While research on white matter connectivity in children with DLD is limited, like other neurodevelopmental populations, there seems to be a connection between language impairment and altered white matter volume and diffusivity (movement of water molecules along white matter pathways). Here, we provide an overview of these findings starting with white matter volume changes in DLD as compared to TD and then we turn attention to the diffusivity of white matter tracts involved in language processing within dorsal and ventral regions.

#### 2.3.1. White Matter Volume

The findings from studies investigating overall white matter volume in children with DLD parallel the findings reported earlier in this paper for gray matter volume, in that results are varied due to differences in methodology and age of participants. However, there is some consistent evidence for children with DLD having overall increased white matter volume [34,36,37]. Similar to the gray matter pattern discovered by Soriano-Mas and colleagues (2009), these researchers discovered that an increase in white matter may be mediated by age. In their 2009 paper, Soriano-Mas and colleagues found that younger children (<12 years of age) with DLD showed an increase in white matter volume, while older children (>12 years of age) did not [33,37]. In contrast to these results, Girbau-Massana et al. (2014) did not find significant differences in white matter volume from their sample of younger children (mean age: 9.4 years) when compared to controls [33]. These difference in findings may be attributed to different methodological approaches. Unlike Soriano-Mas (2009) [37], Girbau-Massana et al. (2014) [33] included measures of intracranial volume in their analysis, which as noted previously, is an important consideration when calculating measures of brain volume. At first glance, the results from Soriano-Mas that link an increase in white matter volume to language impairment in DLD are surprising, since increases in white matter volume have been interpreted as improved cognitive skills across development [80]. In this case, a different interpretation is warranted. Findings of abnormally increased white matter volume in children with DLD may indicate that the underlying microstructure is impacted, which could result in poor connectivity across networks. Thus, in the next section, we turn to the literature investigating white matter integrity using diffusion MRI.

#### 2.3.2. White Matter Diffusivity in Child Language-Impaired Populations

If the transmission of signals between different neural regions is impaired or slowed in any way, it could lead to disruptions of the tightly orchestrated processes needed for successful language processing and production, such as early auditory processing or the production of appropriate grammatical morphemes. Further, it can impact the strength and efficiency of language networks, which could lead to changes in connectivity as well as white and gray matter volume. Diffusion MRI (dMRI; described in more detail in Appendix B, Table A2) is an imaging method that can be used to reveal details about the integrity of white matter pathways by measuring the diffusion of water molecules in brain tissue. This method has been commonly used to track neurodevelopmental changes attributed to normal brain maturation, as well as assess the underlying integrity of these connections in children with neurodevelopmental disorders.

As children mature and new skills are mastered, the brain develops more efficient structural and functional neuronal connections. These changes can be characterized by different diffusion tensor imaging (DTI) indices, such as fractional anisotropy (FA), mean diffusivity (MD), axial diffusivity (AD), and radial diffusivity (RD), that reflect movement and direction of water molecules along fiber bundles over a period of time (see Appendix B for a description of these indices) [79]. In typically developing children, the general expectation is that across development axons become more densely packed and myelinated [82]. This is represented by increases in FA and AD along the primary axis and decreases in MD and RD along the orthogonal axes. Additionally, these indices are expected to exhibit asymmetries across the two hemispheres as different brain regions develop at different rates due to experience. However, in populations with neurodevelopmental language disorders, such as ASD, researchers have found altered patterns of white matter diffusivity in left hemisphere language tracts, which may contribute to language impairment [85,86]. In children with DLD, differences in white matter development may underlie the language impairments observed. The next section of this paper explores dMRI findings along dorsal and ventral language pathways in children with DLD.

#### 2.3.3. Dorsal and Ventral Language Pathways

**Figure 4:**
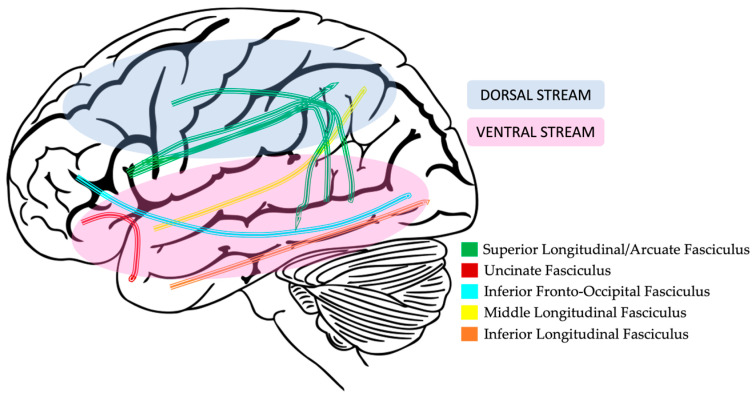
White matter pathways within the dorsal (blue) and ventral (pink) streams. Dorsal stream paths include the superior longitudinal fasciculus (SLF) and arcuate fasciculus (AF). Ventral stream paths include the uncinate fasciculus (UF), inferior frontal occipital fasciculus (IFOF), middle longitudinal fasciculus (MdLF), and inferior longitudinal fasciculus (ILF).

There is an abundance of evidence supporting a dual-stream (dorsal and ventral; Figure 4) model of language processing in the brain. Dorsal stream white matter pathways are thought to map sound to distinct linguistic units, and ventral stream white matter pathways are thought to map sound to meaning [87,88,89]. As described below, some studies have reported differences along these pathways in children with DLD when compared to age-matched, typically developing children.

#### 2.3.4. Dorsal Pathway Findings in DLD

From the few studies that have investigated microstructural brain differences in children with DLD, one of the common findings in the dorsal language pathway is decreased FA in the superior longitudinal fasciculus (SLF) and an increase in either MD or RD in the arcuate fasciculus (AF) [64,90,91]. This pattern is in contrast to what occurs in typically developing children in which as language skills develop, myelin content increases to create more efficient white matter pathways, which is indexed by an increase in FA and a decrease in MD and RD [91]. This lack of change in children with DLD indicates abnormal development of dorsal stream tracts involved in language processing.

#### 2.3.5. Ventral Pathway Findings in DLD

Longitudinal studies of ventral white matter development in typically developing children have indicated that the inferior fronto-occipital fasciculus (IFOF) and inferior longitudinal fasciculus (ILF) develop early on with increases in FA and RD and decreases in AD and MD values throughout childhood and adolescence until they peak in young adulthood. Interestingly, the uncinate fasciculus (UF) does not reach peak values until around 30–40 years of age [79,92]. Even fewer studies have investigated ventral language tracts in children with DLD, but the most consistent findings are decreased FA in the left hemisphere IFOF, UF, and ILF, with some limited evidence of decreased FA in the right hemisphere as well [64,91,93]. Vydrova et al. (2015) also found increases in MD and RD in the left hemisphere IFOF and ILF and a bilateral increase in RD for the UF [91]. This pattern of decreased FA and increased MD and RD in ventral stream language tracts of children with DLD differs from the typical pattern of white matter development. Additionally, in line with reported asymmetry and volume differences in gray matter brain regions, there also seems to be a lack of leftward asymmetry and an increase in volume in the ILF and IFOF [91,93].

In sum, there is converging evidence that suggests that children with DLD have altered micro- and macro-structures within language-related white matter pathways, but the correlation with observed language abilities remains elusive as studies have used a limited range of standardized language assessments, if at all, and as such, only a few have found correlations between language abilities and DTI indices. Importantly, differences in white matter architecture point to a possible contributor to language impairments in DLD. While there is a dearth of evidence for DLD, we can look to findings with other language-impaired populations to direct future investigations of white matter pathway anomalies and their effect on language abilities. Alterations in the development of the architecture supporting different higher-order functions such as language and learning systems could lead to alterations in function of different brain regions, which may in turn contribute to neurodevelopmental disorders. In fact, research with infants at risk for developing autism and dyslexia has revealed that early alterations in white matter pathways can impact developing language abilities among others, as the coordination and function of cortical networks is constrained by the architecture of white matter pathways [82,94,95]. Given the structural alterations reported in gray and white matter regions of the brain in children with DLD, the investigation of functional brain activity during language tasks is warranted. In the next section of this paper, we explore studies investigating functional brain activation patterns in those diagnosed with DLD.

## 3. Functional Neuroimaging Findings in DLD

Functional magnetic resonance imaging (fMRI) provides researchers (and clinicians) a glimpse into the window of the active brain. The examination of brain activity during specific tasks can inform theories of behavior, in this case, language.

### 3.1. Functional Magnetic Resonance Imaging (fMRI) Studies

Studies investigating functional brain activity in children with DLD using fMRI vary significantly across the tasks that are used and the age ranges studied. However, the general picture suggests that children with DLD differ across activation levels (hypo- and hyper-activation), locations, and laterality patterns of brain activation when compared to typically developing children during language-related tasks. Since fMRI results are largely based on task demands, it is important to understand that different tasks will produce different patterns of activation. However, the goal here is to highlight consistent patterns of regional activation differences across the broader categories of expressive and receptive tasks (though see Appendix C for a more detailed review of activation patterns across specific tasks). As will become clear from the discussion below, more studies are needed to not only verify results (particularly across a wider range of tasks) but to also explore whether differences in the level, location, or laterality of activation patterns are due to other factors such as maturational changes (as discussed in prior sections).

#### 3.1.1. Regional Activation Patterns for Expressive Language Tasks

Across the different expressive tasks (covert/overt naming, nonword repetition, etc.) employed in the DLD fMRI literature, one consistent finding is either a lack of activation or reduced activation in the left IFG and unexpected activation of the right IFG (see Table A3 in Appendix C for an overview) [38,96,97,98]. Reduced activation in the left and increased activation in the right is consistent with reports of a lack of leftward asymmetry and altered gray matter volume within this region. It is difficult to speculate on how altered gray matter and reduced activation are related given that studies differ as to the directionality of volumetric differences. On one hand, if the size of the left IFG is larger in those with DLD, then perhaps reduced activation reflects a lack of neural pruning and less efficient neural networks. On the other hand, if the size of the left IFG is smaller, then it may reflect inadequate neural architecture (including possible aberrant white matter connectivity) to support the carefully sequenced operations necessary for expressive language tasks, which may explain the recruitment of the right IFG as a compensation mechanism. In fact, individuals with post-stroke aphasia have demonstrated this pattern of right hemisphere homologue recruitment following left IFG damage in both the acute (<6 months post-stroke) and chronic (>6 months post-stroke) stages, suggesting that when there are alterations to brain structure, homologue regions may take over function in an attempt to compensate [99,100].

Other noteworthy DLD findings include altered activation in the superior temporal gyrus/sulcus (STG/STS) where the planum temporale is located and an increase in subcortical activation in both hemispheres [38,96,97,98]. Regional gray matter findings indicate that these regions also show patterns of asymmetry and volumetric differences which may be related to altered activation patterns, but the exact nature of this connection is unclear. Whatever the case, findings of structural and functional alterations to these distributed language regions would support accounts of aberrant language abilities in DLD, but more work is needed to understand the directionality of the neuroimaging differences. Furthermore, given the possibility of maturational effects and the variability across those diagnosed with DLD, studies employing larger sample sizes should investigate whether there are phenotypic differences in structure and function of these regions.

#### 3.1.2. Regional Activation Patterns for Receptive Language Tasks

The results from studies that used receptive language tasks (passive listening, implicit learning, etc.) produced fewer consistencies than those from the expressive language tasks, likely due to the lack of overlap in tasks employed (see Table A4, Appendix C). However, in receptive language imaging studies, children with DLD demonstrated different patterns of activation in the left (and in one case, right) STG/STS where the planum temporale is located [101,102,103]. As discussed above, the planum temporale is generally larger in the left hemisphere than in the right in typically developing populations, but children with DLD do not evince this same pattern of volumetric asymmetry, nor do they show typical levels of activation in this region. Like with previous regions discussed, it is difficult to speculate on the relationship between these structural alterations and functional outcomes, but there does seem to be a connection between the aberrant structure and function of the planum temporale in DLD. These findings are consistent with theoretical accounts of auditory processing deficits (discussed below), but abnormalities in the neural architecture of those diagnosed with DLD extend beyond this early auditory processing region, so more work is needed to understand how developmental differences in the planum temporale impact language function more broadly.

To summarize, limited evidence suggests that regions that consistently show structural alterations also show functional alterations (i.e., IFG, planum temporale, and subcortical structures such as the caudate), but again, findings vary in terms of level (hyper- or hypo-activation) and location of activation patterns. While some of the variability can be attributed to differences in methodology, these results may also point to differences in the underlying properties that support brain function (e.g., neurovascular, hemodynamic, and other neurobiological processes). As described in more detail below, fMRI studies rely on capturing changes in the concentration of deoxygenated blood in the brain, which is based on underlying assumptions about the amount and rate of cerebral blood flow (CBF) to these critical brain regions. However, the underlying assumptions about CBF (and its relationship to the fMRI signal) might not be appropriate when evaluating brain activation in children with DLD [104]. If CBF deviates from typical patterns in children with DLD, particularly in language-related brain regions, it may over- or under-estimate the functional contributions of those regions. Furthermore, if blood flow to a brain region is not enough to sustain functionality (but is enough to sustain viability of the tissue), it could contribute to adverse language outcomes. Therefore, the last section of this paper looks at CBF patterns by exploring neuroimaging findings in DLD.

### 3.2. Cerebral Blood Flow Patterns

There is a tight coupling between neuronal activity and increased blood flow to active brain regions (e.g., neurovascular coupling). When neurons are active, they send chemical and electrical signals to blood vessels to dilate which in turn increases the flow of blood and allows an abundance of oxygen and glucose to be delivered to neurons as a fuel source. As neurons consume the influx of oxygen, the blood becomes deoxygenated. It is this change in oxygen content on which the fMRI or blood oxygen level dependent (BOLD) signal is based [105]. However, if the relationship between neural activity and the BOLD response is not accurately modeled via the hemodynamic response function (HRF), then activation patterns may be misinterpreted. Prior research has revealed that the HRF may deviate from the canonical pattern with clinical populations [106]. For example, following a stroke, the amount of time it takes for blood to perfuse neural tissue (i.e., transit delay time) may be longer than normal and the amount of blood that gets delivered to neural tissue may be reduced [99,107,108]. Thus, when conducting fMRI studies with suspected neurologically compromised populations, including neurodevelopmental groups, it is critical that the modeled HRF reflects the true nature of blood flow in the brain in order to extract accurate BOLD signal estimates.

#### Cerebral Blood Flow Patterns in DLD

The studies reported in this section utilized different methods to measure cerebral blood flow (CBF) including single-photon emission computed tomography (SPECT), positron emission tomography (PET), and transcranial Doppler (TCD) ultrasound. Each method relies on different mechanisms to capture blood flow patterns, and all come with inherent advantages and disadvantages, which may underlie discrepant results. Nonetheless, these methods have provided some insight into potential differences in CBF patterns between children with DLD and typically developing children.

One of the most common findings in studies utilizing SPECT is abnormal hemispheric lateralization of blood flow patterns and/or hypoperfusion (i.e., reduced CBF) in left hemisphere language regions in DLD [109,110,111,112,113]. Briefly, Lou et al. (1990) found that children with language impairment exhibited reduced CBF in the left perisylvian region while at rest compared to the right hemisphere, while Tzourio et al. (1994) found a lack of left hemisphere blood flow during a phonemic discrimination task [111,113]. Another common SPECT/PET finding is differences in blood flow (as indexed by glucose metabolism) in subcortical regions [112,114,115]. For example, Hwang et al. (2006) found a pattern of reduced CBF in the right basal ganglia and left globus pallidus, while Im et al. (2007) found decreased blood flow in the thalamus [114,115]. These subcortical findings are in alignment with other studies previously discussed here that report differences in volume and activation in these regions (e.g., [35,96,98]). Subcortical regions, such as the basal ganglia, are thought to work in conjunction with regions of the brain that are known to support functions such as language learning [68,73,114]. In children with DLD, altered blood flow patterns to these linguistic and non-linguistic regions of the brain may ultimately impact language abilities.

Though there is a fair amount of agreement in findings across these early SPECT and PET studies, it should be noted that because these approaches require an injection of a radioactive isotope, the control groups were children with other clinical disorders such as ADHD, cluster headaches, or muscular dystrophy. Therefore, more studies are needed that use better validated measures that are less invasive, so as not to limit comparison groups to those diagnosed with clinical disorders.

Unlike SPECT and PET, transcranial Doppler (TCD) does not require the injection of a radioactive contrast agent and thus has been relied on in the past for CBF investigations in DLD. Using this method in conjunction with a functional task (e.g., fTCD), Whitehouse and Bishop (2008) reported abnormal patterns of CBF in young adults (mean age: 18.5) with DLD, characterized either by relatively greater right hemisphere lateralization or bilateral cerebral blood flow (indicating a lack of typical left hemisphere asymmetry) [116]. However, these results failed to be replicated in a younger sample of children with DLD (6–12 years). In typically developing populations, CBF increases across early childhood until it reaches a peak around 7–10 years of age [117,118]. Following this peak, CBF declines with age, though it remains relatively stable through early and middle adulthood [119,120]. It is possible that CBF, which is greater in childhood, may not follow the typical trajectory into adulthood in those with DLD, leading to differences in laterality. One other possibility for these reported differences is that, as a method, fTCD is highly operator dependent and it is limited to larger, basal arteries such as the middle cerebral artery (which supports a large number of language regions), and thus, it cannot be used to measure changes in blood flow to areas of the brain that are supported by smaller blood vessels [121]. Therefore, future studies investigating CBF patterns in DLD should use methods that are less user-dependent and invasive, such as MRI arterial spin labeling. Additionally, more studies across a range of ages would help elucidate differences in maturational CBF patterns that may underlie changes in the BOLD signal. By ensuring that the assumptions about the physiological properties of the BOLD signal are met, it may help to bridge disparate functional imaging findings in DLD, which in turn would help with better connecting imaging findings to theoretical accounts of DLD.

## 4. Interim Summary: Neuroimaging Patterns in DLD

Thus far, we have highlighted similarities as to the structural and functional differences in DLD as compared to TD across neuroimaging studies, while acknowledging that findings are based on limited evidence and often have contrasting results (likely due to methodological differences). We approached this portion of the overview by reporting converging evidence across structural (whole brain, gray matter, white matter, etc.) and functional measures (task-based activation and cerebral blood flow). The current state of the DLD neuroimaging literature suggests that *structurally*, when compared to typically developing children, children with DLD have smaller overall brain sizes, they show differences in whole brain and regional gray and white matter volume (potentially mediated by age), and they have altered white matter macro- and micro-structure of language-related tracts. *Functionally*, they demonstrate differences in the level of brain activation (i.e., hypo- and hyper-activation), the location of activation (i.e., regional differences), and laterality of activation (i.e., left vs. right hemisphere recruitment). They also show differences in the level and lateralization patterns of cerebral blood flow.

Importantly, while using a different approach, we identified similar regions of altered brain structure and function (planum temporale, inferior frontal gyrus, and caudate nucleus) to a prior systematic review of neuroimaging studies in DLD conducted by Mayes and colleagues (2015) [16]. While it is clear from both this overview and the review conducted by Mayes et al. (2015) that altered brain structure and function are important components of DLD pathology, the connection between neuroimaging findings and observed language deficits has been less apparent, as research findings from neuroimaging studies are often reported independently of theoretical research findings. Therefore, it is our goal in this paper to begin to make those connections so that moving forward as a field we can design more theoretically informed neuroimaging studies with more sophisticated linguistic material to better tease apart how aberrant brain structure and function relates to the range of reported language impairments in DLD.

In the next section, we briefly review selected theoretical accounts of DLD, and after each section, we comment on the potential link between the proposed theoretical account and the neuroimaging findings discussed above in an attempt to elucidate brain–behavior relationships.

## 5. Neuroimaging Evidence Supporting Theoretical Accounts of Language Impairment Patterns in DLD

In order to illustrate how neuroimaging evidence can better inform theoretical accounts of language impairment patterns in DLD, we now discuss some common theories that have been proposed to explain language impairment patterns in DLD.

Across the published studies, there are a range of theoretical accounts that attempt to describe and explain observable language error patterns in children with DLD. While errors are part of typical language development (overgeneralizations, pronoun resolution, etc.), here we refer to error patterns that do not resolve with time [122]. Theories outlining these error patterns can generally be divided into three well represented arguments in the literature that point to deficits specific to (1) linguistic knowledge (e.g., morphosyntax), (2) domain general language processing (e.g., phonological working memory, speech perception, etc.), or (3) non-linguistic cognitive processes associated with language (e.g., working memory, processing speed, etc.) [9]. In isolation, each of these theoretical approaches have merit; however, children with DLD can exhibit both linguistic and non-linguistic impairments, making it difficult to propose a single comprehensive and encompassing theory that can account for hallmark deficits, such as difficulty with grammar, while also explaining other less consistent findings, such as deficits in attention and speed of processing. It is our hope that by making connections between theoretical accounts of DLD and the more consistent neuroimaging findings, that we can point to potential areas to focus future research endeavors. To accomplish these goals, below we present three theoretical accounts of language impairment in DLD. After each account, we integrate the imaging findings described above so as to build a bridge between two seemingly disparate areas.

### 5.1. Theoretical Approach: Linguistic Knowledge

Deficits in morphology and syntax (i.e., morphosyntax) are ubiquitous in children diagnosed with DLD. These observations have led to proposals suggesting that language deficits stem from limitations in linguistic knowledge (e.g., tense marking rules, phrase structure rules, movement, etc.; Table 2). One of the earliest among the **agreement, tense, and number marking** accounts is the Extended Optional Infinitive (EOI) account [123]. According to Wexler (1994), around 4–5 years of age, typically developing children undergo a stage by which they optionally mark the tense and number on finite verbs in main clauses (e.g., she drinks coffee can optionally be *she drink coffee; the asterisk represents an ungrammatical utterance) [124]. In cases where tense/number is not marked, children tend to produce the infinitive form of the verb (i.e., drink). Building off of Wexler’s account, Rice et al. (1995) suggested that children with DLD remain in this optional infinitive stage for a period of time that *extends* beyond that of a typically developing child, before their grammatical usage catches up to that of an adult (if it does at all; see [125]).

The EOI account works well to characterize error patterns made by children diagnosed with DLD when speaking languages such as English and French, but it fails to account for the grammatical errors made by children who use other languages such as Italian and Spanish due to cross-linguistic differences in syntactic structure. Thus, a number of theoretical accounts followed which expanded on the EOI premise that children with DLD struggle with aspects of tense and agreement (see Table 2) [125,126,127]). However, evidence has shown that children with DLD are not limited to errors in just tense and/or agreement as these accounts suggest, and thus, this theory underspecifies observed errors. To address this issue of limited scope, other accounts focused on **structural complexity**, such as the Representational Deficit for Dependent Relations (RDDR), suggesting that deficits stem from difficulty with performing these complex operations. One example would be the movement of a wh-question word (e.g., who) to the front of a sentence to form a question (Table 2) [122,128,129]. Though accounts such as the RDDR cover a wider range of deficits, especially across languages, they lack specificity, particularly concerning how and in which instances children with DLD struggle with complex operations. Further, the connection between deficits across different linguistic domains (i.e., syntax, morphology, and phonology) needs clarification since it is unclear how deficits in processing complex syntactic structures would also result in other deficits discussed in the sections below.

Other theories that focus on the **application of rules**, like the Narrow Rule Learning account [130], posit that children with DLD tend to stick to structures that have a high number of exemplars in the language, which may help bridge theoretical gaps, but no single linguistic theory can explain all of the inconsistencies in deficits across linguistic domains [122]. In addition, the majority of linguistic accounts have focused on observable production errors in individuals with DLD, which limits the ability to generalize to language deficits as a whole.

**Table 2:** A summary of theoretical accounts of DLD that describe deficits in linguistic knowledge. Error types are based on common groupings of described patterns but do not represent an exhaustive list of all theories proposed to date.

Error Type	Theoretical Account
Agreement/Tense/Number Marking	Extended Optional Infinitive (EOI) Account [123] Agreement/Tense Omission Model (ATOM) [125] Extended Unique Checking Constraint (EUCC) [126]
Structural Complexity	Representational Deficit for Dependent Relations (RDDR) [128] Computational Grammatical Complexity (CGC) Account [131]
Application of Rules	Narrow Rule Learning Account [130]

#### Neuroimaging Evidence Supporting Linguistic Knowledge Theories

Though the source of grammatical deficits (or whether there is even a single source) is unclear in DLD, there is no doubt that children with DLD struggle with the grammatical features of language [132]. Interestingly, the type of grammatical impairments observed are often specific to the individual language spoken by the child, suggesting that both genes and the environment play a role in the abnormal development of language regions in the brain in DLD [132]. There is significant debate surrounding the neural regions that support grammatical learning, but two of the structures mentioned earlier in this overview, the inferior frontal gyrus (IFG) and the caudate, are often implicated [133]. In fact, Tagarelli et al. (2019) linked the left and right IFG regions as well as the caudate to learning the grammatical features of an artificial language in adults [133]. Common theories outside of grammatical learning have suggested that Broca’s area (the pars opercularis and pars triangularis regions of the IFG) is involved in processing syntactically complex sentences, such as those with long-distance dependencies [61,134], comprehending sentences that have high working memory demands [62], and articulatory rehearsal to support comprehension [60].

In children with DLD, it may be the case that the aberrant development of the IFG impacts comprehension and production of the grammatical features of the language being acquired. Given that the precise functional role of the left IFG is still unclear and structural findings in DLD are mixed, it is difficult to do more than speculate on the link between linguistic accounts of DLD and structural brain findings. However, overall increases or decreases in activation of the IFG as well as differences in size suggest altered function of the region and may further indicate altered microstructural architecture, such as changes in neuronal density and/or network connectivity, which impact language processes. For example, the left IFG has connections with the basal ganglia, specifically the caudate and putamen (known collectively as the striatum), through cortico-striatal circuits, a finding that has been supported via fMRI, diffusion, and functional connectivity studies [135]. These circuits may influence the learning of grammatical rules of the language spoken. Aberrant connections between the caudate and IFG may lead to an overpopulation of neurons (due to a lack of neuronal pruning), which would increase total gray matter volume and impact functionality. Alternatively, reductions in gray matter could be associated with neurotransmitter dysregulation or altered dendritic morphology, which may also impact the function of the regions. Whatever the case, there does seem to be growing evidence for alterations to the IFG and caudate that impact language abilities. However, as mentioned, grammatical deficits are a large component of DLD, but they are not the only feature of language affected. As a result, other theoretical accounts, discussed below, have suggested that the source of deficits stem from broader problems with either linguistic (but non-grammatical) or non-linguistic, cognitive systems.

### 5.2. Theoretical Approach: Language Processing Accounts

Linguistic-based accounts of DLD, such as those described above, benefit from being able to explain specific error patterns in language use; however, they often fail to encompass the wide range of deficits across languages. Further, they may not accurately reflect how a child with DLD processes (as opposed to produces) language. Therefore, some theories posit that language impairment stems from aberrant processing in domain general language systems that impact language use (Table 3). Among these theories are those that suggest that children with DLD have general auditory processing deficits. In a seminal series of studies in the 1970s, Tallal and Piercy [136,137], found that children with language impairment (those diagnosed with DLD and those with hearing impairments) performed worse than TD control children on a variety of tone and speech perception tasks that included categorization, discrimination, and temporal sequencing of auditory information. Children with DLD in particular seemed to struggle with auditory stimuli that were presented briefly or in rapid succession, which led the authors to suggest that the source of deficit stems from difficulties with **temporal processing** at the phoneme level.

In an attempt to expand on Tallal and colleague’s supposition of an underlying temporal processing deficit, Schwartz, Scheffler, and Lopez (2013) argued that children with DLD have deficits in **perceptual processing**, specifically, categorical perception and the use of perceptual speech cues, which in turn affects storage and access to lexical representations [138]. Though these perceptual accounts can be compelling, one point of weakness is in describing how they can lead to disruptions of grammatical processing as opposed to just lexical or phonological processing. Leonard, McGregor, and Allen (1992) attempted to address this limitation by proposing the Surface Account, which posited that the complex operations required to process grammatical markers taxes the system resulting in incomplete processing of grammatical morphemes [139]. It is argued that this difficulty is then further amplified by the low perceptual saliency of grammatical morphemes, which increases the amount of exposure needed to learn them. However, evidence in support of this theory is mixed (see [122] for a review); thus, as it stands, more work needs to be carried out to understand if and how these processing differences result in the range of deficits observed.

**Phonological short-term memory** (also referred to as phonological working memory) is another commonly cited source of language difficulty for children with DLD. Different models of how language is stored have been proposed, but the most prominent view of phonological short-term memory comes from Baddeley and colleagues [140,141,142]. Based on the model, Gathercole and Baddeley (1989) proposed that phonological short-term memory supports vocabulary development by helping to form stable phonological representations [143]. This proposal was supported by findings that demonstrated a relationship between poor nonword repetition skills and smaller vocabularies in children with DLD, indicating that they struggle to form and store stable phonological representations [144]. Thus, nonword repetition tasks have become a consistent and reliable measure for characterizing what many have suggested are phonological short-term memory impairments in children with DLD, but again, these theories are unable to account for observable impairments at the syntactic level during production and they tend to lack explanatory power for comprehension impairments [145]. Though there are processing theories that extend beyond the phoneme and word-level, the majority of studies have focused on this level. Thus, to better account for the full range of deficits observed in children with DLD, it is suggested that researchers look to other processing theories that have been proposed for other language-impaired populations as they may inform a larger range of observed deficits [146,147].

**Table 3:** A summary of theoretical accounts of DLD that describe deficits in language processing. Error types are based on common groupings of described patterns but do not represent an exhaustive list of all theories proposed to date.

Error Type	Theoretical Account
Temporal Processing	Attending to rapid or brief auditory stimuli (e.g., phoneme level) [136,137]
Perceptual Processing	Phoneme discrimination/categorization [138] Surface Account [139]
Phonological Short-Term Memory	Lexical selection/access (poor nonword repetition) [145] Unstable phonological representations (smaller vocabularies) [143,144]

#### Neuroimaging Evidence Supporting Language Processing Theories

Prior research has shown that a white matter tract connecting regions around the auditory cortex in the temporal gyrus to the motor regions in the frontal lobe can be reconstructed in newborns using diffusion MRI, suggesting that the ability to map acoustic information to linguistic representations is already present from birth [148,149]. However, it is not until later in childhood that the pathway between the temporal lobe and inferior frontal gyrus is detectable, suggesting that dorsal pathway language tracts, involved in processing complex sentences, develop later in life [148]. Any alterations to language tracts present at birth may contribute to altered structure and function of the neural regions that support language processing as a child learns and grows.

Structural abnormalities of the planum temporale support theoretical accounts that posit that children with DLD have deficits related to temporal processing [136,137] and speech perception [138]. A reduction in left planum temporale gray matter may indicate that the microstructural architecture (densely packed cortical columns) needed to support rapid temporal processing is anomalous in individuals with DLD. This may also impact connections to other language regions such as the connection between the temporal lobe and IFG. In fact, it has been shown that both the IFG and regions in the superior temporal sulcus (STS) are highly connected hubs within the language processing network; thus, early differences within these regions may lead to language processing deficits [150].

The IFG has also been implicated in phonological short-term memory and phonological processing, which could account for the phonological deficits described in the section above [46]. Indeed, some studies with children with DLD have found differences along these tracts in terms of DTI metrics (mainly FA and MD), volume, and lateralization patterns when compared to typically developing children, but the correlation with language abilities has been more difficult to elucidate. Though there seems to be compelling neurological evidence in support of language processing theories, some researchers have proposed that DLD is not a disorder specific to the language system, but rather affects broader cognitive domains, which in turn have an impact on language-specific processes. Similar theories have been proposed with other language-impaired populations, but additional investigations are needed to understand the precise nature of deficits in DLD.

### 5.3. Theoretical Approach: Non-Linguistic Cognitive Processing

The notion that children with DLD have broader processing limitations beyond language has received considerable attention in the DLD literature (Table 4). In looking at the literature from other well-studied language-impaired populations, namely individuals with aphasia, it has been proposed that linguistic knowledge remains intact in these individuals, but the ability to tap into the cognitive resources necessary to build the representations or perform complex operations with them are impaired (though see [151] for an opposing view) [152]. Studies investigating these limitations generally focus on aspects of working memory, such as resource allocation capacity and attention. Within the DLD literature, similar proposals have been investigated.

For example, Montgomery (2000) studied the ability of children with DLD to allocate resources during a word recall task [153]. Based on the assumptions of the working memory model proposed by Just and Carpenter (1992), the premise of their study was that people have a limited pool of resources by which they can process and store incoming information and children with DLD have even greater reductions in processing capacity [154]. They found that children with DLD could complete simple processing tasks, but as the task complexity increased (i.e., dual-load tasks where participants had to sort items by size and semantic category), performance worsened compared to controls, suggesting that children with DLD have reduced resources (e.g., processing capacities), which constrained the ability to successfully perform multiple, simultaneous cognitive operations. One big limitation of processing capacity theories is that it is not always clear what the “resource” being allocated is, though it is often conceived as an attentional one [141]. The problem with this is that children with DLD do not always demonstrate impairments in attention, or if they do, it tends to be situational. Further, it is challenging to design studies that can be falsified given that processing capacity is difficult to distinguish and measure.

Other non-linguistic cognitive theories have suggested that children with DLD are slower to perform a wide range of linguistic and non-linguistic tasks based on evidence from reaction time studies [122]. One early account for **reduced processing speeds** was the Generalized Slowing Hypothesis, which posits that children with DLD perform more slowly on both linguistic and non-linguistic tasks when compared to typically developing peers, and this difference is proportional to the complexity (i.e., the number of operations) required by the task [155]. Like with attentional studies, the problem with this hypothesis is that not all children with DLD show slowed reaction times on non-linguistic tasks, and those that do seem to do so selectively across different tasks [156]. Therefore, more work is needed to understand what causes some children to have slower processing speeds than others, as well as whether it affects broader, domain general neural systems or more specific ones.

One final group of domain-general cognitive theories discussed here is related to **impaired learning systems** [72]. The most comprehensive account of learning deficits in DLD is the Procedural Deficit Hypothesis (PDH) [73]. According to the PDH, children with DLD have a deficit in the procedural memory system. This system is comprised of frontal and subcortical brain regions that support the learning of linguistic, motor, and other non-linguistic sequences. The PDH posits that aberrant function of the procedural system can lead to impairments in any of the systems associated with it; thus, the PDH attempts to account for the wide range of linguistic and non-linguistic impairments reported in the DLD literature. It also makes a distinction between procedural and declarative systems. The procedural system is thought to underlie the acquisition and use of rule-governed grammatical computations, while the declarative system is thought to underlie storage of lexical knowledge, such as semantic features and the memorization of more arbitrary, word-specific information like irregular past-tense verb forms. Research investigating sequence-based procedural learning tasks has found that children with DLD demonstrate impairments in this realm but are less likely to show deficits in non-sequenced based declarative tasks [72]. However, Ullman and Pierpoint (2005) point out in their model that the procedural and declarative systems are not entirely independent of one another due to their connections with other systems, such as those involved in working memory, so it is possible for children with DLD to show deficits in processes supported by both systems [73]. This becomes particularly relevant when trying to account for smaller vocabularies in children with DLD, since the declarative system likely supports this process.

Though the PDH provides a nice framework for capturing one of the most striking impairments in DLD (grammatical errors), more work needs to be done to refine the hypothesis, particularly in terms of the functional boundaries between the procedural and declarative systems. Further, this model provides one of the most comprehensive accounts for the varied neuroimaging findings, but in an effort to account for such a broad range of deficits, the model posits that any deficit can be explained by the procedural system or connections to it, with limited evidence to support these claims.

**Table 4:** A summary of theoretical accounts of DLD that describe deficits in non-linguistic cognitive processing. Error types are based on common groupings of described patterns but do not represent an exhaustive list of all theories proposed to date.

Error Type	Theoretical Account
Reduced Resource	Reduced Processing Capacity [153]
Reduced Processing Speed	Generalized Slowing Hypothesis [155]
Impaired Learning System	Procedural Deficit Hypothesis (PDH) [73]

#### Neuroimaging Evidence Supporting Non-Linguistic Cognitive Processing Theories

Returning to the Procedural Deficit Hypothesis along with other theories of language learning and sequencing, the caudate has been identified as a locus of language learning deficits in DLD. This is the case for other language-impaired populations, such as those with aphasia as well [72,73,157]. As discussed previously, it is not clear if altered neurophysiology leads to language learning impairments or if language impairments impact the growth and development of neurons in DLD, which in turn affects learning, but subcortical circuits seem to underlie some aspects of language learning and there are reports of regional differences in these areas, so additional research is needed.

Further, theories that posit a general slowing of both linguistic and non-linguistic cognitive systems could be supported by more research investigating microstructure architecture along these pathways. For example, a number of studies have indicated that children with DLD have decreased FA along left hemisphere language tracts. FA is thought to index axonal microstructure (diameter and density), myelination concentration, and degree of fiber crossings [158]; thus, a decrease in FA may signify less efficient transmission of neural signals, which may lead to overall slower processing. However, it remains an open question whether reports of slowed processing in DLD are truly language-specific or more general, especially given the high degree of connectivity the language network has with other networks such as those involved in learning, attention, and memory. Studies investigating structural and functional connectivity patterns in DLD along with measures of tract integrity (e.g., FA, MD, RD, etc.) may help to answer these questions.

### 5.4. Interim Summary: Linking Theory to Brain

There are a number of different accounts that have been proposed in an attempt to explain patterns of language impairment in children with DLD. We divided these theories into three well-represented categories, namely deficits in linguistic knowledge, language processing, and non-linguistic cognitive processing, and briefly discussed evidence supporting each. Each of these theories does well at capturing specific measured behaviors that differ in DLD compared to typically developing peers, but when zooming out more broadly to examine the range of behaviors reported (e.g., tense omission, poor phonological awareness, slower processing speed, etc.), there are a number of competing accounts that in isolation are not able to explain the broad scope of impairments in DLD. On one end of the theoretical continuum, the linguistic accounts lack explanatory power and are too narrow in scope, especially since most tend to focus on word-level, production errors; and on the other end, the more general non-linguistic processing accounts are too broad, as they cannot explain why language (and grammar) is affected above and beyond other cognitive systems in DLD if the deficit is in a domain-general system. One possibility is that DLD is a spectrum disorder (like autism) with different phenotypes [11]. If this is the case, neuroimaging evidence could help with the identification of different phenotypic groups by linking specific neural profiles to behavioral patterns. This would require much larger datasets than are typical in DLD research and would be most informative with longitudinal designs.

Alternatively, another approach could be to examine language abilities that are more commonly affected in children with DLD as a starting point to elucidate the relationship between altered brain structure/function and variable language profiles. For example, difficulty with the use of grammatical features of language and poor performance on nonword repetition tasks are common in DLD. Both of these abilities in typically developing individuals have been linked to the inferior frontal gyrus (IFG) [43,130,131]. In a recent study by Bahar and colleagues (2023) with children with DLD, the authors found that performance on a nonword repetition task was a significant predictor of surface area within the left IFG [41,46,133,134]. It may be the case that alterations within the left IFG are linked to difficulties with both of these language tasks. Further, given that the IFG is a densely connected hub within the language network, abnormal development of the region could impact other connected regions, such as the superior temporal sulcus. This would help explain empirical evidence indicating that language deficits exist across different language domains, as certain deficits could be linked back to a common neural substrate, and variations beyond those deficits could potentially be linked to alterations in structural and functional connections with other regions.

One other possibility is that the underlying neurobiological mechanisms supporting brain development are impacted in DLD. Recent work from Bahar et al. (2023) and Krishnan et al. (2022) is beginning to explore this area by investigating the underlying properties of brain tissue [41,74]. For example, as mentioned previously, Krishnan et al. (2022) measured gray matter myelin content in children and adolescence with DLD and found less myelin in the caudate nucleus and left inferior frontal gyrus. Together, these brain regions partially comprise the corticostriatal loop, which plays a role in sequential learning tasks such as learning the grammatical rules of a language. Based on their findings, the authors posited that children with DLD have difficulty learning the complex rules of language, as evinced by altered myelin content within this language learning circuitry (though they do not speculate on the causal direction of the relationship between reduced myelin and language abilities). Typical myelin development begins in utero and is subject to a number of carefully timed genetic processes that slowly give way to more environmental influences postnatally [76]. Thus, a reduction in myelin content could be related to specific genetic markers early on in DLD. Then, as a child continues to develop, experience could have a greater impact on the development of higher-order cognitive functions, like language learning. This idea is supported by the fact that myelination within the brain regions associated with these higher-order functions takes longer in comparison to sensory and motor regions to develop. This could help provide support for more domain-general theories, like the Procedural Deficit Hypothesis or the Generalized Slowing Hypothesis mentioned in Section 5.3 and Section 5.4. Unlike sensory and motor cortices, the brain regions involved with language processes take longer to “mature”; thus, early alterations to the underlying properties supporting brain function could have a detrimental impact on later-developing language network architecture, thus resulting in the range of language impairments that we observe in DLD.

## 6. Discussion: The Current State (and Limitations) in Linking Theory to Brain

It is clear that more work needs to be conducted to obtain a better sense of the neural organization and networks engaged in language processes in children diagnosed with DLD. Overall, the current state of the field suggests that children with DLD have atypical brain volume, laterality, and activation/connectivity patterns compared to their neurotypical peers. Behaviorally, one of the most striking impairments is in the production of grammatical morphemes, but research has demonstrated impairments in a range of other linguistic and non-linguistic tasks, such as vocabulary development, nonword repetition, and short-term memory (though it should be noted that it is difficult to create purely non-verbal short-term memory tasks). In terms of neuroimaging research, across both structural and functional brain studies, the planum temporale (located in the posterior superior temporal gyrus), the inferior frontal gyrus, and the caudate nucleus consistently show altered patterns in children with DLD when compared to typically developing children. Taken together, it is reasonable to speculate that atypical language development in DLD is not an environmental side effect but is in fact related to anomalous development of brain structures and/or function. However, few studies have attempted to link theoretical accounts of language impairment in DLD to neuroimaging findings, resulting in two disparate bodies of literature.

The goal of this overview was to synthesize the literature in a way that could help lay the groundwork for more theoretically motivated neuroimaging research. Though the connections made here are purely speculative based on the evidence presented, they do align well with the recent literature demonstrating structural and functional differences in the corticostriatal pathways that support language learning [41,72,74,159]. These recent studies utilize more consistent methodological approaches to make connections between altered brain structure and theoretical accounts of DLD, and they explore the underlying properties that support broader measures of structural brain development beyond brain volume (i.e., myelin, surface area, cortical thickness, etc.). This is important because prior neuroimaging research with children with DLD rarely overlaps in methodological approaches and often focuses on these gross brain measures, making it difficult to draw conclusions about the underlying contributors of altered brain structure or function in DLD. At this point, more research with larger sample sizes and carefully defined participant criteria is needed to replicate previous neuroimaging findings and build on our current knowledge of language impairment patterns in DLD. By laying a foundation for more theoretically motivated neuroimaging research, we may be able to better link neural differences to specific language profiles that align with current theoretical accounts of language deficits in DLD.

## 7. Future Directions: New Approaches in Linking Theory to Brain

There were a number of ways to craft this overview. We chose to categorize aspects of studies based on a variety of measures used to evaluate brain structure (i.e., whole brain volume, gray matter volume, white matter diffusivity, etc.) and brain function (i.e., task-based activation and cerebral blood flow). Our goal was to present converging evidence about neural regions implicated in DLD in order to link theory to brain. Importantly, using this approach, we found a similar pattern of results to a systematic review conducted by Mayes and colleagues (2015) in which they divided neuroimaging studies in DLD by the methodology employed (e.g., semi-automatic morphometry, voxel-based morphometry, etc.). Similarly to our findings, Mayes et al. (2015) reported that the posterior superior temporal gyrus, the inferior frontal gyrus, and the caudate are implicated in DLD pathology [16]. Given the consistencies across these reviews and more recent studies in DLD, future research will benefit from exploring the relationship between altered structure/function of these brain regions and performance on language tasks that are linked to activation within those regions.

A problem discussed throughout this overview, though, is the lack of consistency across studies in terms or the direction of volumetric differences (e.g., larger in the left hemisphere, smaller in the right, etc.) and the location and level of brain activation in the three brain regions frequently implicated in language impairment in DLD. One possibility is that broad measures of brain structure (i.e., volume) and brain function may overshadow important underlying properties that contribute to these gross measures. For example, as previously mentioned, Bahar et al. (2023) demonstrated that altered brain volume in DLD was largely driven by differences in surface area rather than cortical thickness, which is noteworthy as surface area and cortical thickness follow distinct developmental trajectories and are likely driven by distinct neurobiological mechanisms [41]. By exploring the underlying properties that index developmental changes, it can help to better account for inconsistent findings within the literature.

To date, one area of neuroimaging research that has received little attention is the processes underlying neuronal function and connectivity, particularly within the language network. Research from another language-impaired population, individuals with aphasia, has demonstrated that reduced cerebral blood flow to middle temporal regions correlates with auditory comprehension impairments, despite these regions appearing structurally uncompromised by the lesion [99]. It may be the case that important language regions, such as the left inferior frontal network, are hindered during development due to aberrant blood flow, underconnectivity, etc., resulting in those consistently observed morphosyntactic deficits in DLD. By examining distributed networks and the processes that underlie neuronal function (e.g., cerebral blood flow, glucose metabolism, etc.) in children with DLD (as compared to neurotypically developing children) it may help to further bridge theoretical accounts as well as aid in the identification of potential biomarkers of DLD.

## Figures and Tables

**Table 1:** Language problems commonly reported in children with DLD. Linguistic categories and descriptions provided are based on the principal dimensions of language difficulty outlined in the CATALISE-2 report [15]. Children with DLD can present with a range of these characteristics, though difficulties with syntax and morphology are ubiquitous in DLD.

Phonology	Syntax/Morphology	Word Finding/ Semantics	Pragmatics/Language Use	Discourse	Verbal Learning/Memory
Linguistic (not motor) difficulties with phonology Phoneme substitutions and deletions Poor phonological awareness	Difficulty using grammatical features of language (e.g., subject–verb agreement) to convey meaning Difficulty interpreting meaning conveyed by grammatical markers (e.g., past tense -ed in English)	Word finding difficulties (may use non-specific words, substitutions, circumlocutions, or avoid topics) Limited vocabulary use and knowledge of word meanings	Literal interpretations and difficulty with abstract concepts Insufficient contextual information when conversing Less aware of social cues Prosodic aberrancies in intonation and stress	Disjointed utterances Difficulty linking sentences and understanding broader topics	Reductions in verbal short-term memory capacity Difficulty with statistical pattern recognition

## Data Availability

Not applicable.

## Appendix A. Referenced DLD Neuroimaging Studies

**Table A1:** Summary of studies investigating neural differences in children with DLD using structural and functional neuroimaging methods. SLI = specific language impairment; TD = typically developing; LI = language impaired; DLD = developmental language disorder; ALI = autism + language impairment; ALN = autism + language normal; IFG = inferior frontal gyrus; LN = language normal; PT = planum temporale; ASD = autism spectrum disorder; VBM = voxel-based morphometry; CSF = cerebral spinal fluid; WM = white matter; GM = gray matter; L = left; R = right; STS = superior temporal sulcus; STG = superior temporal gyrus; ROIs = regions of interest; MTG = middle temporal gyrus; FA = fractional anisotropy; SLF = superior longitudinal fasciculus; HSL = history of speech and language; PTG = posterior temporal gyrus; DLI = developmental language impairment; ANL = autism + normal language; LH = left hemisphere; RH = right hemisphere; IFOF = inferior fronto-occipito fasciculus; ILF = inferior longitudinal fasciculus; AF = arcuate fasciculus; UF = uncinate fasciculus; ECFS = extreme capsule fiber system; BA = Brodmann area; SMG = supramarginal gyrus; NL = normal language; DSD = developmental speech disorder; SMA = supplementary motor area; TPJ = temporal parietal junction; ADHD = attention deficit hyperactive disorder.

Reference	N (M/F), Mean Age (SD or Range)	Method	Key Findings in DLD (Compared to TD)
**Structural Imaging Studies**			
Plante et al., 1991 [160]	SLI: 8 (8 M), 5.2 months TD: 8 (8 M), 4.2–9.6 months	Manual Morphometry	Children with SLI had significantly larger right hemisphere perisylvian volumes and left hemisphere volumes were as expected, showing abnormal asymmetry. No significant differences were found for extra-perisylvian regions between SLI and TD due to individual differences.
Jernigan et al., 1991 [57]	LI: 20 (13 M/7 F), 8.9 (±0.7) TD: 12 (8 M/4 F), 9.0 (±0.7)	Semi-automatic and Manual Morphometry	Children with LI had significantly smaller volumes in the L posterior perisylvian region compared to the R region and significantly greater volume in the R prefrontal regions compared to the L region. The authors found weak statistical evidence for reversed asymmetries in the perisylvian region (right > left).
Preis et al., 1998 [58]	DLD: 21 (14 M/7 F), 8.33 (±21 months) TD: 21 (14 M/7 F), 8.25 (±21 months)	Manual Morphometry	There were no differences in the planum temporale or the planum parietal asymmetry between DLD and TD children. DLD children had smaller forebrain sizes, which accounted for smaller planum temporale bilaterally in DLD.
Gauger et al., 1997 [32]	SLI: 11 (8 M/3 F); 9.0 (±2.3) TD: 19 (14 M, 5 F), 8.9 (±1.6)	Manual Morphometry	The pars triangularis was significantly smaller in the L hemisphere of children with SLI. Children with SLI were more likely to have rightward asymmetry of language structures.
Herbert et al., 2003 [36]	DLD: 24 (16 M/8 F), 8.31 (5.7–11.3) TD: 9.1 (6.5–11)	Semi-automatic and Manual Morphometry	Total brain volume was greater in children with DLD as compared to controls. A significant main effect of sex was found, with males showing significantly larger total brain volumes as compared with females. Regional differences between DLD and control children revealed cerebral white matter volume was 13% larger in the DLD sample.
De Fossé et al., 2004 [59]	ALI: 16 (16 M), 9.8 (±2.1) ALN: 6 (6 M), 8.3 (±0.9) SLI: 9 (9 M), 9.9 (±2.3) TD: 11 (11 M), 10.4 (±2.7)	Semi-automatic and Manual Morphometry	Non-language-impaired individuals had larger cerebral volumes than language-impaired individuals (both SLI and ALI). The IFG was larger on the R side in LI and larger on the L side in LN participants. The PT was equal in size for language normal participants and larger in the L for language-impaired individuals.
Herbert et al., 2005 [66]	DLD: 15 (15 M) ASD: 16 (16 M) TD: 15 (15 M) Total: 46 between 5.7 and 11.3 years.	Semi-automatic and Manual Morphometry	Cerebral symmetry patterns in children with DLD and ASD were similar but differed from TD patterns. In both autistic and DLD samples, there was a substantial increase compared with controls in the aggregate amount of cortical parcellation unit (PU) asymmetry. The ASD and DLD brains showed an increase in the number (and volume) of cortical PUs with rightward asymmetry, but at the same time, they showed either no loss (ASD) or only a small loss (DLD) in the number (and volume) of cortical PUs with leftward asymmetry.
Soriano-Mas et al., 2009 [37]	SLI: 36 (24 M/12 F), 10.58 (±2.80) TD: 36 (24 M/12 F), 10.88 (±2.83)	VBM	**Global Volume Results:** Children with SLI showed larger gray and white matter volumes than controls, but no differences were observed in CSF spaces. Global gray matter and white matter was increased in children with SLI when compared to TD children, but this finding was not consistent in the older children subgroup. **Regional Volume Results:** Children with SLI had increased gray matter in the R perisylvian region and the occipital petalia, in the L middle occipital gyrus. Findings indicated that in younger children, several regions of gray matter increase in SLI (entorhinal area bilaterally, and the temporopolar cortex, the caudate nucleus, the motor-precentral cortex, and the precuneus of the left hemisphere). There was a specific volume increase in the left middle occipital gyrus for children with SLI. The R perisylvian region, in comparison to controls, was increased during the whole age range (i.e., no interaction between gray matter volume and age), and so was the left temporopolar cortex, with a specific volume increase in younger children with SLI (i.e., interaction between gray matter volume and age). Significant volume increases were observed in younger children with SLI in the juxtacortical WM of the right medial frontal cortex and bilaterally in the middle temporal gyrus. There were no notable findings in the older group in regard to WM.
Badcock et al., 2012 [38]	SLI: 10 (9 M/1 F), 13.5 (±2.6) SLI-Sibling (SIB): 6 (4 M/2 F), 18.0 (±3.8) TD: 16 (7 M/9 F), 12.5 (±4.7)	VBM	Total GM volume did not differ. DLD had more GM than TD in L IFG, R insula, and L intraparietal. DLD has less GM than TD in pSTS bilaterally, R STG, R caudate, and R side of midbrain at substantia nigra, medial frontal polar cortex, R medial superior parietal, and L Occipital. DLD had more GM than SIB in L anterior intraparietal. There was less GM in the R parietal opercular and L occipital. SIB compared to TD, with more GM in ventral extent of central sulcus and retro splenial area bilaterally. SIBs had less GM in bilateral caudate, R putamen, R medial geniculate, and L fusiform than TDs.
Girbau-Massana et al., 2014 [33]	SLI: 10 (6 M/4 F), 8.5–10.9 (6 of the SLI participants had SLI + Reading Disorder or RD) TD: 14 (10 M/4 F), 8.2–11.8	VBM	Children with SLI had significantly lower overall GM volume, especially at the right postcentral parietal gyrus, and greater CSF volume. Children with SLI and RD exhibited a significant smaller volume of GM in right postcentral parietal gyrus than children with TD. The CSF volumes were significantly greater in children with SLI and RD and SLI than in children with TD. Ratios of gray to white matter were not significantly different in children with SLI and RD when compared to children with TD.
Lee et al., 2020 [64]	2 cohorts of subjects: **Cohort 1:** DLD: 14 (5 M/9 F), 22.42 (±2.02) TD: 12 (4 M/8 F), 22.10 (±0.51) **Cohort 2:** DLD: 19 (8 M/11 F), 16.96 (±1.49) TD: 61 (25 M/36 F), 16.62 (±1.40)	Semi-Automatic Morphometry and Diffusion Tensor Imaging (DTI)	**Dorsal Pathway:** The DLD group had significantly larger relative volumes of the L transverse temporal gyrus in the dorsal pathway than the comparison group. None of the other ROIs in the dorsal pathway showed significant age or group effects. **Ventral Pathway:** The ROIs comprising the ventral pathway showed significantly larger volumes for DLD in the R pars orbitalis, of the R temporal pole, and the L temporal pole for DLD subjects. Significant increases in relative volumes were seen across age for the L MTG, and for the L pars triangularis in DLD. **DTI:** Significantly reduced FA in the DLD group compared with the comparison group was found in the L and R SLF. There was an age-by-group interaction effect on the R SLF only for the comparison group. Significantly reduced FA values were found in the DLD group for the L IFOF and left UF. DLD group had no significantly increased FA across age in the L ILF and R UF (although the controls showed increased FA here). The DLD group also had smaller intracranial volumes.
Krishnan et al., 2022 [74]	DLD: 33 (22 M/11 F), 12.48 (±1.80) HSL: 20 (17 M/3 F), 12.40 (±1.67) TD: 56 (28 M/28 F), 12.41 (±1.62)	MPM Quantitative Image (Myelin and Iron Concentration), and T1-Weighted structural	Lower myelin content in bilateral caudate nuclei and L IFG. No group differences were found for iron concentration.
Bahar et al., 2023 [41]	DLD: 54 (39 M/15 F), 12.4 (±0.25) TD: 74 (41 M/33 F), 12.6 (±0.2)	T1-Weighted Structural (Surface Area, Cortical Thickness, and Gray Matter Volume)	**Surface Area:** DLD showed smaller surface area bilaterally in the IFG extending to the anterior insula, in the posterior temporal and ventral occipito-temporal cortex, and in portions of the anterior cingulate and superior frontal cortex. **Cortical Thickness:** No differences in cortical thickness, or asymmetry of these cortical metrics. **Gray Matter Volume:** DLD showed smaller volumes in the R pars triangularis, L MTG, and PTG.
Lee et al., 2013 [35]	DLI: 12 (4 M/8 F), 21.99 (±1.56) TD: 12 (4 M/8 F), 22.06 (±0.51)	Semi-Automatic Morphometry and Diffusion Tensor Imaging	The DLI group showed smaller bilateral caudate nucleus, globus pallidus, and bilateral thalamus, as well as smaller cerebral lobes including the occipital lobe and frontal lobe. The DLI group was significantly larger than the control group in the relative volumes of the hippocampus, putamen, the R globus pallidus, and the nucleus accumbens. The DLI group showed lower FA values in the cerebral lobes including the frontal and occipital lobes (no significant findings for other ROIs).
Roberts et al., 2014 [90]	SLI: 14 (8 M/6 F), 9.73 (±2.69) ALI: 16 (14 M/2 F), 9.80 (±2.57) ANL: 18 (16 M/2 F), 11.47 (±3.25) TD: 25 (16 M, 9 F), 11.42 (±2.92)	Diffusion Tensor Imaging	Both ASD and LI were associated with elevated mean diffusivity of the LH arcuate fasciculus. There was a main effect of ASD on the axial, but not radial, component of diffusivity, and a main effect of LI on the radial, but not axial, component of diffusivity, suggesting that although ASD plus LI and SLI may share a microstructural anomaly of elevated radial diffusivity, they differ in axial diffusivity. No differences were found in L arcuate fasciculus FA for SLI or ASD. A main effect of ASD was found on axial diffusivity but not radial diffusivity; conversely, there was a main effect of LI on radial diffusivity but not axial diffusivity. There was also a significant ASD vs LI interaction on axial diffusivity. No group differences were observed in the RH arcuate fasciculus for any of the DTI measures.
Vydrova et al., 2015 [91]	SLI: 37 (25 M/12 F), 8.4 (±1.6) TD: 34 (18 M/16 F), 8.9 (±1.6)	Diffusion Tensor Imaging	Children with SLI had reduced FA of the investigated tracts, and corresponding increases in mean diffusivity values (AF bilaterally, L IFOF and L ILF), increases in radial diffusivity component (AF bilaterally, L IFOF, L ILF and UF bilaterally), and decreases in axial diffusivity component (R IFOF and L UF). Children with SLI showed bilaterally larger volumes of the ILF compared to controls.
Verly et al., 2019 [93]	DLD: 17 (11 M/6 F), 10.07 (±2.01) TD: 22 (15 M/7 F), 11.00 (±1.11)	Diffusion Tensor Imaging	**DTI Metrics:** Participants with DLD showed decreased FA values in the L ILF and middle longitudinal fascicle (MdLF). A higher tract volume of the SLF, MdLF, and the longitudinal posterior part of the superior longitudinal fascicle (SLF) was detected in children with DLD compared to TD children. Also, lower apparent diffusion coefficient (ADC) values of the ILF and a higher FA of the SLF were detected in children with DLD. There was a pattern towards increased RH FA values in patients with DLD relative to controls across the SLF, SLF_anterior, MdLF, ILF, and ECFS. A trend towards lower ADC values was observed in children with DLD relative to controls across the SLF, SLF_longitudinal, SLF_posterior, and all ventral language-related WM tracts. Finally, a trend towards a higher tract volume was observed for all dorsal and ventral WM tracts in patients with DLD. **Lateralization:** TD children showed LH lateralization of FA for all language-related WM tracts, except for the posterior part of the SLF and the ECFS. A significant RH lateralization was found for the anterior part of the SLF. None of the studied dorsal and ventral language-related WM tracts showed a significant L or R lateralization pattern, except for the MdLF in children with DLD.
**Functional Imaging Studies**			
Liegeois et al., 2003 [96]	KE Family Affected: 5 (3 M/2 F), 34.8 (±17.0) Unaffected: 5 (3 M/2 F), 25.3 (±3.4)	fMRI Covert and Overt Verb Generation and Word Repetition	**Overall:** Affected members showed significant under-activation bilaterally in Broca’s area and the putamen. **Covert Task:** The distribution of activation was the same for the unaffected and control group in L IFG, but for affected members, there was more diffuse involvement of RH compared to LH and posterior compared with anterior bilaterally. **ROIs:** Under-activation for affected most notably in L BA44, but also R putamen/globus pallidus, L SMG, R IFG, and L precentral gyrus. Bilateral overactivation for affected in pSTG, L precentral, R temporal pole, and a number of parietal ROIs. **Overt Task:** Activation was mainly bilateral. There was activation in L IFG and bilateral putamen. No significant overactivation was detected. **Repetition Task:** Significant overactivation in L BA44 and L anterior insula.
Hugdahl et al., 2004 [101]	SLI: 5 (2 M/3 F), 11–70 years TD: 6 (2 M/4 F), 15–61 years	fMRI Passive Listening	Reduced activation in MTG and STS. Unlike TD, SLI also activated areas in the R inferior frontal lobe.
Weismer et al., 2005 [161]	DLD: 8 (5 M/3 F), 13.10 (±6 months) TD: 8 (6 M/2 F), 14.0 (±7 months)	fMRI Encoding and Recognition Tasks	No significant differences in activation patterns or laterality. There were two distinct activations in the IFG (BA44/45) and the insular portion of BA44. **Encoding Task:** Significant difference in precentral sulcus and parietal for encoding. DLDs showed hypoactivation in the insular portion of IFG during recognition. Less coactivation between IFG and STG during encoding. DLD had significant correlations between activation in parietal and frontal memory ROIs and parietal and STG during encoding. **Recognition Task:** DLD had a weak association between the STG and frontal ROIs and STG and parietal.
Dibbets et al., 2006 [103]	SLI: 6 (all male); 6.10 (±8.4 months) TD: 7 (all male), 6.10 (±6.3 months);	fMRI Switch Task	**Non-Switch Task:** Increased activation in L and R frontal area and superior parietal and occipital lobe. **Switch Task:** Increased activation in L/R frontal, temporal, and cingulate regions.
De Guibert et al., 2011 [97]	SLI: 21 (9 M/12 F), 11.4 (±3.3) TD: 18 (9 M/9 F), 12.7 (±3.0)	fMRI Lexico-Semantic Tasks (category fluency and responsive naming) and two Visual Phonological Tasks	**Category Fluency Task:** Lack of left lateralization in BA44 and reduced activation. **Naming Task:** More activation in RH, with no activation in some of the regions that TDs activated. Insula was bilateral for SLI, whereas it was only left-lateralized for TD. L hypoactivation in posterior STG/SMG. **Minimal Pair Triplets Task:** SLI showed RH activation, but TD did not. There was R hyperactivation in the insula. **Segmentation Triplets Task:** SLI showed no activation in the ROIs that were expected (IFG, STG, SMG, or insula). Lack of L lateralization in BA44. Overall, there was a lack of lateralization in the LH, with no differences in lateralization of the rest of the brain. The L posterior STG/SMG was hypoactivated, while the R anterior insula was hyperactivated.
Badcock et al., 2012 [38]	SLI: 10 (9 M/1 F), 13.5 (±2.6 years) SLI-Sibling (SIB): 6 (4 M/2 F), 18.0 (±3.8) TD: 16 (7 M/9 F), 12.5 (±4.7)	fMRI Overt Word Generation and Passive Listening Tasks	TD and SIB showed a similar pattern of activation. DLD showed no activation in L IFG like other groups and much lower levels of activation in bilateral STG. TD and SIB showed L lateralization for speech but DLDs had less (due to a couple of individuals) in frontal and temporal areas. **Structure–Function Relationship:** Increased GM and decreased activation in inferior frontal region for DLDs and decreased GM and activation in posterior temporal region.
Plante et al., 2017 [102]	LI: 16 (7 M/9 F), 20.0 (±2.2 years) NL: 16 (7 M/9 F), 20 (±3.5 years)	fMRI Implicit Language Learning Task	There were no differences in the location of activation, but the level of activation was increased in L BA44, L SMG, L STG.
Pigdon et al., 2020 [98]	DLD: 18 (10 M/8 F), 124 months (±3) DSD: 15 (5 M/10 F), 123 months (±4) TD: 42 (22 M/20 F), 123 (±6) TD-matched subset: 17 (8 M/9 F), 123 (±5)	fMRI Nonword Repetition Task	DLD and DSD groups primarily activated subcortical regions (thalamus and globus pallidus), while TDs primarily activated cortical regions that are similar to adult activation patterns for nonword repetition, namely bilateral STS/STG, anterior insula, L IFG, SMA, precentral gyrus and motor cortex, L TPJ, anterior cingulate, and thalamus. However, there were no regions that survived thresholding when comparing the two groups; thus, there were no statistically significant differences.
Krishnan et al., 2021 [159]	DLD: 50 (35 M/15 F), 12.0 (10–15) TD: 67 (38 M/29 F), 12.1 (10–15)	fMRI Verb generation task	No activation differences in the L IFG or putamen and no lateralization differences. Sub-threshold activation differences in bilateral caudate and L IFG in a subset of children with poor task performance.
**Cerebral Blood Flow (CBF)**			
Denays et al., 1989 [110]	Dysphasia: 14 (10 M/4 F), aged 5–16 years	SPECT	**Expression Deficit:** Hypoperfusion in frontal convolution of LH (Broca’s area). **Comprehension and Expression Deficits**: Hypoperfusion in the L tempo-parietal and hypoperfusion in upper and middle R frontal lobe. Three children with comprehension/expression deficits showed no abnormality. And 11 out of 14 were hypoperfused in language regions; there was hypoperfusion in areas for expressive deficits only, but for those with both, there was nothing in L IFG.
Lou et al., 1990 [111]	24 patients (median 10 yr, range 6–15) all from special school and with antenatal and perinatal events. ADHD: 9 ADHD + Phonological-syntactic dysphasia: 8 Severe phonological syntactic dysphasia: 3 Severe verbal decoding issues: 3 Severe lexical-semantic: 1	SPECT	For the language-impaired group, differences were less obvious than in other groups, but overall CBF was lower in the L perisylvian region than the R. For the phonological-syntactic only group, CBF to L prefrontal was lower than R.
Tzourio et al., 1994 [113]	Expressive–Receptive: 7 (4 M/3 F), 10 (±1) Expressive: 7 (6 M/1 F), 8 (±1) ADHD: 6 (6 M), 8 (±1)	SPECT	There was an absence of LH activation during phonemic discrimination task in children with expressive–receptive deficits compared to those with only expressive difficulties and those with ADHD. Abnormal lateralization patterns were found for language in the more severely impaired group.
Chiron et al., 1999 [109]	Dysphasia: 8 (8 M), 10.2 Duchenne Muscular Dystrophy: 8 (8 M), 10.7	SPECT	**Dichotic Task:** Lack of activation in Broca’s but CBF increase in R homologue compared to Duchenne Muscular Dystrophy group. **Dichaptic Task:** Bilateral increase in CBF as opposed to only in RH for Duchenne group.
Ors et al., 2005 [112]	SLI: 19 (11 M/8 F), 9.7 (7–15) ADHD: 12 (11 M/1 F), 9.4 (7–14)	SPECT	Children with SLI had lower CBF values in R parietal and subcortical regions and no L asymmetry as is typical in development.
Hwang et al., 2006 [114]	DLD: 21 (14 M/7 F), 4.2 (±0.8) Tension Headaches: 17 (13 M/4 F), 11.0 (±1.9)	SPECT	Reduced CBF patterns were found in the R putamen, R inferior parietal cortex and the L globus pallidus (though this region did not survive correction for multiple comparisons). There were no regions of increased CBF in DLD compared to controls.
Im et al., 2007 [115]	DLD: 17 (15 M/2 F), 4.7 (±2.1) Split into 2 groups: >5 years and <5 years. ADHD: 10 (9 M/1 F), 7.0 (±1.8)	PET	**>5 Years:** Abnormal findings in the thalamus, cerebellum, basal ganglia, caudate nucleus, and left temporal lobe. **<5 Years:** Abnormal findings in the thalamus, basal ganglia, cerebellum, caudate nucleus, and frontal lobe. Overall, the children with DLD showed decreased glucose metabolism in the frontal, temporal, and right parietal areas.
Whitehouse & Bishop, 2008 [116]	SLI: 11 (7 M/4 F), 18.15 (±3.75) SLI-History: 9 (7 M/2 F), 18.33 (±14.33) ASD: 11, (10 M/1 F), 19.98 (±5.03) TD: 11 (7 M/11 F), 20.88 (±3.29)	Functional Transcranial Doppler (fTCD) Ultrasound Covert and Overt Word Generation	The SLI-History, ASD, and TD groups all demonstrated greater activation in the LH compared to the RH during the task, indicating typical LH dominance for language. The SLI group demonstrated an altered pattern of either RH dominance or bilateral activation.

## Appendix B. Diffusion Tensor Imaging (DTI) Indices

**Table A2:** A description of different measures of diffusivity and common findings in clinical populations compared to individuals with DLD.

Measure	What Is It Measuring?	Clinical Findings
Fractional Anisotropy (FA)	Amount of diffusion along primary axis (axon) compared to secondary and tertiary axes (perpendicular to the axon). FA increases as anisotropy increases (between 0 and 1; [102]). FA may reflect axonal microstructure (diameter and density), myelination concentration, and degree of fiber crossings [109].	**Clinical Populations:** Decreased FA values in patients with neurodegenerative diseases, such as Alzheimer’s Disease and in normal aging [107,109]. **DLD:** The evidence is mixed, but findings suggest decreased FA in the superior longitudinal fasciculus and left hemisphere ventral (see Figure 4) tracts. There is limited evidence for decreased FA in the right hemisphere [62,83,110,111,112].
Mean Diffusivity (MD)	Average diffusion across all three axes (λ_1_ + λ_2_ + λ_3_/3) [108]. MD may reflect structural integrity of axons [107].	**Clinical Populations:** Increased MD in patients with disturbed structural integrity such as those with epilepsy and chronic stroke [107,113]. **DLD:** Increased MD in the arcuate fasciculus [112,114].
Axial Diffusivity (AD)	Amount of diffusion along the primary axis (λ_1_) [108]. AD may reflect axonal integrity/damage [115].	**Clinical Populations:** Decreased AD in patients with axonal degeneration and inflammation, such as individuals with multiple sclerosis (MS) [104,116]. **DLD:** No evidence of changes in AD. More research is needed.
Radial Diffusivity (RD)	Average diffusion perpendicular to the primary axis (λ_2_ + λ_3_/2) [108]. RD may reflect myelin integrity/damage [115].	**Clinical Populations:** Increased RD in patients with myelin loss, such as individuals with MS [113]. **DLD:** Increased RD in the arcuate fasciculus [112,114].

## Appendix C. Regional Activation Patterns by Task

**Table A3:** Differences in network activation patterns for expressive language tasks in individuals with DLD when compared to typically developing populations. Blue boxes indicate regions where activation patterns were significantly different, and the text indicates the type/directionality of the differences found. Not significant (N.S.) indicates regions that were activated for both groups but in which no significant differences in activation patterns were found. Dashed lines indicate regions that were not investigated for either group in the study. STG/S = superior temporal gyrus/sulcus; GP = globus pallidus; PT = planum temporale; RH = right hemisphere.

		Left Hemisphere Regions							Right Hemisphere Regions	
Task	Reference	Inferior Frontal Gyrus (IFG)	Non-IFG Frontal	Supramarginal/Angular Gyrus	Precentral Gyrus/ Sulcus	Superior Temporal Gyrus/ Sulcus	Middle/ Inferior Temporal Gyrus	Sub-Cortical	IFG	Sub-Cortical
**Covert Naming**	Liegeois et al., 2003 [96]	Hypo-activation	N.S.	Hypo-activation	Hyper-activation	Hyper-activation	Hyper-activation	N.S.	Hypo-activation	Hypo-activation (putamen/ GP)
	De Guibert et al., 2011 [97]	Hypo-activation	N.S.	Hypo-activation	N.S.	Hypo-activation (PT)	N.S.	-----	Activation found (IFG and Insula)	-----
	Badcock et al., 2012 [38]	No activation found	-----	-----	-----	Hypo-activation	-----	N.S.	Activation found	Hypo-activation (putamen)
**Overt Naming**	Liegeois et al., 2003 [96]	Hypo-activation	N.S.	-----	N.S.	N.S.	N.S.	Hypo-activation (putamen)	N.S.	Hypo-activation (putamen)
	Krishnan et al., 2021 [159]	N.S.	N.S.	Hyper-activation (at lowered voxel threshold only in RH)	-----	N.S.	-----	Hyper-activation (in caudate at lowered voxel threshold in low-performing subset)	N.S.	Hyper-activation (in caudate at lowered voxel threshold in low-performing subset)
**Nonword Repetition**	Pigdon et al., 2020 [98]	No activation found	No activation found	No activation found	No activation found	No activation found	-----	Hyper-activation (thalamus/ GP–uncorrected)	No activation found	Hyper-activation (thalamus/ GP–uncorrected)

**Table A4:** Differences in network activation patterns for receptive language tasks in individuals with DLD when compared to typically developing populations. Blue boxes indicate regions where activation patterns were significantly different, and the text indicates the type/directionality of the differences found. Not significant (N.S.) indicates regions that were activated for both groups but no significant differences in activation patterns were found. Dashed lines indicate regions that were not investigated for either group in the study. STG/S = superior temporal gyrus/sulcus; PT = planum temporale; MTG = middle temporal gyrus; AG = angular gyrus.

		Left Hemisphere							Right Hemisphere		
Task	Reference	Inferior Frontal Gyrus (IFG)	Non-IFG Frontal	Supra-marginal/Angular Gyrus	Precentral Gyrus/ Sulcus	Superior Temporal Gyrus/ Sulcus	Middle/ Inferior Temporal Gyrus	Sub-cortical	IFG	Sub-cortical	Other
**Passive Listening**	Hugdahl et al., 2004 [101]	-----	-----	-----	-----	Hypo-activation (PT)	Hypo-activation	-----	N.S.	-----	Hypo-activation (PT, MTG)
**Implicit Learning**	Plante et al., 2017 [102]	Hyper-activation	N.S.	Hyper-activation	N.S.	Hyper-activation	-----	-----	-----	-----	N.S. (STG/S, MTG)
**Verbal Working Memory**	Weismer et al., 2005 [161]	Hypo-activation (insular portion)	N.S.	-----	Hypo-activation (parietal)	N.S.	-----	-----	N.S.	-----	-----
**Executive Control**	Dibbets et al., 2006 [103]	-----	Hyper-activation (medial, orbito, superior)	-----	N.S.	Hyper-activation	Hyper-activation	Hyper-activation (cingulate)	-----	Hyper-activation (cingulate)	Hyper-activation (intraparietal, AG)
